# COVID-19: A Chimera of Two Pandemics

**DOI:** 10.1017/dmp.2020.223

**Published:** 2020-06-25

**Authors:** Parnian Jabbari, Forouq Jabbari, Saied Ebrahimi, Nima Rezaei

**Affiliations:** Research Center for Immunodeficiencies, Children’s Medical Center, Tehran University of Medical Sciences, Tehran, Iran; Network of Immunity in Infection, Malignancy and Autoimmunity (NIIMA), Universal Scientific Education and Research Network (USERN), Tehran, Iran; Maternal, Fetal and Neonatal Research Center, Tehran University of Medical Sciences, Tehran, Iran; Research Department of Rajaei Heart Center, Iran University of Medical Sciences, Tehran, Iran; Department of Immunology, School of Medicine, Tehran University of Medical Sciences, Tehran, Iran

**Keywords:** COVID-19, health policy, pandemics, vaccination

The first cases of coronavirus disease (COVID-19) were observed in China in late 2019 and developed to a pandemic in a matter of weeks due to high rates of person-to-person transmission.^1,2^ Public authorities soon responded to the crisis by placing stay-in-shelter orders. Although effective in reducing the transmissions, the challenges posed by these rules urged their termination. As individuals are re-engaging in social activities, given that an effective vaccine may not be available before 2021^3^ makes us rely on slowing person-to-person transmission to control this pandemic. Although due to similar routes of transmission, we often refer to the H1N1 pandemic of 1918 to model how COVID-19 will evolve, we believe that the still ongoing HIV/AIDS pandemic can inspire us in 3 main ways to fight the COVID-19 pandemic in a similar way that the HIV/AIDS pandemic was controlled, despite growing globalization.

## ADDRESSING HIGH-RISK GROUPS

In early stages of the HIV/AIDS pandemic, intravenous drug users and individuals with high-risk sexual activity were noted to be high-risk.^4^ Global organizations emphasized on the importance of destigmatization, acknowledging individuals’ choices, and breaking the vicious cycle of discrimination leading to the susceptibility for the disease and the disease being a source of discrimination.^4^

In the case of COVID-19, the elderly and those with comorbidities are high-risk and advised to self-isolate. Apart from the psychological effects,^4^ such restrictions are in contrast with enabling individuals’ choices. Authorities need to provide guidelines for communities to enable social involvement of high-risk individuals while providing them with a safe environment.

## EDUCATIONAL MEASURES

A safe environment in terms of contracting COVID-19 cannot be achieved unless every individual is well educated on preventive measures. Current guidelines emphasize on physical distancing, hand-hygiene, and the use of personal protective equipment (PPE) to slow person-to-person transmission.^4^ Thus far, the emphasis of countries has been on providing more testing and reports on mass education of preventive measures, and monitoring adherence to these guidelines are sparse, if any.

Education is shown to be a key factor in bringing the HIV/AIDS pandemic under control. Although the long timeframe for HIV made much of this education possible, we can still rely on the effectiveness of these methods by using mass media and interactive educational programs.^5^

## ELIMINATING HEALTH INEQUALITIES

Although HIV/AIDS was first spread among well-connected networks, many factors, including discrimination and marginalization, shifted the burden of the disease on less privileged parts of the society.^4^ This led to worldwide attempts to provide high-risk groups with protection equipment such as condoms, clean needles, and free access to antiretroviral therapy.

In the COVID-19 pandemic, these risk factors translate to lack of access to soap and clean water and the inability to social distance due to low socioeconomic status and living conditions. These are effective and cheap tools to control the spread of many infectious diseases, including COVID-19. High transmission rates of the disease warrant calls for global action to provide PPE to all social groups indiscriminately.^2^ These measures can help bring under control COVID-19 – a pandemic chimera of the HIV/AIDS and H1N1 pandemics.
